# Visual Outcomes and Higher Order Aberrations Following LASIK on Eyes with Low Myopia and Astigmatism

**DOI:** 10.2174/1874364101812010084

**Published:** 2018-05-31

**Authors:** Smita Agarwal, Erin Thornell, Chris Hodge, Gerard Sutton, Paul Hughes

**Affiliations:** 1 Wollongong Eye Specialists, 13 Market st, Wollongong, Australia; 2 University of Wollongong, Northfields ave, Wollongong, Australia; 3 Vision Eye Institute, 8-10 Woniora rd, Hurstville, Australia; 4 University of Sydney, Camperdown, Australia

**Keywords:** LASIK, Aberrations, Contrast sensitivity, Vision quality, Myopia, Astigmatism

## Abstract

**Background:**

Laser-Assisted *in situ* Keratomileusis (LASIK) can induce corneal aberrations that can impact vision and patient satisfaction. Recent developments in laser technologies have helped minimise these aberrations.

**Objective:**

To assess the quality of vision and change in Higher-Order Aberrations (HOAs) following wavefront-optimized LASIK in low-myopic astigmatic patients.

**Methods:**

LASIK was performed on a total of 76 eyes in patients with myopia <4.0 D and cylinder <2.0 D using the WaveLight® EX500 excimer and FS200 femtosecond laser platform. Visual acuity, contrast sensitivity and HOAs were measured at 1 and 3 months postoperatively and compared to preoperative values. Subjective quality of vision was assessed pre- and postoperatively using a VF14 questionnaire.

**Results:**

Mean postoperative Spherical Equivalent (SE) was -0.09 ± 0.26 µm with 95% of patients within ± 0.5 D of attempted SE. Postoperative uncorrected distance visual acuity was 20/20 or better for 96% of patients. Contrast sensitivity increased against horizontal and vertical gratings at all spatial frequencies except for vertical gratings at 18 cycles/degree. Spherical aberration and total HOA increased by 0.085 µm and 0.13 µm respectively. The mean VF14 score increased from 89.2 ± 16.7% to 99 ± 1.4% postoperatively.

**Conclusion:**

LASIK performed using the WaveLight® EX500 excimer and WaveLight® FS200 laser platform provided improved contrast sensitivity and visual acuity with minimal introduction of HOAs, making it a suitable platform for low myopic astigmatic patients.

## 1 INTRODUCTION

Since its first reported use on human eyes by Pallikaris *et al.* [1], LASIK has become the most common refractive procedure to correct ametropia. Current protocols often utilise a dual-laser platform, utilising a femtosecond laser for flap creation and an excimer laser for corneal reshaping, helping refractive surgeons achieve excellent visual and safety outcomes.

Surgeons generally report high levels of patient satisfaction following LASIK, and this generally is dependent on factors such as good postoperative Unassisted Distance Visual Acuity (UDVA). However, some issues that affect patient satisfaction have been identified including residual ametropia and Higher-Order Aberrations (HOA).

Pre-existing HOAs can be exacerbated by LASIK as a result of the ablation process itself as well as multiple other factors including corneal dehydration [2] and decentration [3-5]. The main contributing aberrations include vertical and horizontal coma, Spherical Aberration (SA) and trefoils, which are known to cause visual artefacts such as glare, starbursts and haloes [6]. Visual distortion also results from the induction of an oblate corneal shape. This is particularly problematic following the use of conventional ablation profiles, which are calculated based on the sphere, cylinder and pupil size of the eye. Attempts to obtain a more prolate corneal shape and to minimise HOA induction led to the development of the wavefront ablation systems.

Wavefront-optimized ablation systems reduce HOA induction and visual distortion by utilising population aberration statistics to calculate an ablation pattern that produces a corneal shape that is as close to prolate as possible. Wavefront-guided ablation was later developed to incorporate the patient’s individual preoperative HOA profiles. These systems have been shown to minimise the increase in HOAs in eyes that have undergone LASIK [7-10]. In conjunction, increased speed associated with higher frequency laser and eye tracking systems reduce corneal dehydration and subject fatigue, simultaneously improving postoperative results and patient experience.

The WaveLight^®^ Allegretto Wave^®^ Eye-Q excimer laser has a high laser power (500 Hz), fast eye tracking, high pulse frequency and can be used for both wavefront-optimised and wavefront-guided treatments.

This study aims to assess the outcome of LASIK utilising the WaveLight^®^ EX500 excimer and FS200 femtosecond laser platform, in order to determine the viability of this platform for use on patients with low myopia and astigmatism.

## 2 MATERIALS AND METHODS

This study was undertaken according to the tenants of the Declaration of Helsinki. Ethical approval was provided by the Human Research Ethics Committee of the University of Sydney, Australia.

### 2.1 Inclusion and Exclusion Criteria

A total of 76 eyes from 38 patients between 20 and 40 years of age and with myopia ˂4.0 D and cylinder ˂2.0 D were recruited for the study. The study cohort was predominantly female (68.4%) with an average age of 30.5 ± 5.38 years. Exclusion criteria included patients with a corneal thickness of less than 500 µm, refractive error outside treatable ranges, significant dry eye, irregular astigmatism, keratoconus, refractive instability and concurrent ocular or uncontrolled systemic disease. Patients were advised to not engage in sports such as wrestling, boxing or martial arts during the postoperative period to prevent flap complications.

### 2.2 Preoperative Examination

Preoperative examination included the following: Visual acuity (logMAR UDVA, Corrected Distance Visual Acuity (CDVA); CP-400 vision chart, Optos, Dunfermline, Scotland), refraction (subjective and mydriatic refraction), tear break up time, eye dominance, intraocular pressure (Goldmann applanation tonometer, Takagi, Nakano, Nagano, Japan), corneal thickness (DGH Ultrasound, DGH Technology, Inc., Exton, PA, USA), fundus examination, pupil size (Colvard Pupilometer, Oasis Medical, Glendora, CA, USA), corneal topography (Oculus Pentacam HR, Oculus, Erlangen, Germany and WaveLight-Allegro topolyzer Vario, Alcon Labs, Ft. Worth, TX, USA), contrast sensitivity (CS; CP-400 vision chart, Optos, Dunfermline, Scotland) and HOAs (Oculus Pentacam HR, Oculus, Erlangen, Germany). Quality of vision was measured using a VF14 questionnaire.

### 2.3 Surgery

All procedures were performed by the same surgeon (PHH) at Vision Eye Institute in Hurstville, Australia. Oxybuprocaine Hydrochloride 0.4% (Minims, Chauvin Pharmaceuticals Ltd., Kingston-Upon-Thames, England) topical anaesthetic drops were applied prior to the procedure for all patients. Corneal flaps were created bilaterally using a WaveLight^®^ FS200 laser (Alcon Labs, Ft Worth, TX, USA). Flaps were positioned at a 70° side-cut angle with a superior hinge. Flap thickness was 120 µm and diameter was calculated based on preoperative astigmatism; 9.1 mm was used for eyes with astigmatism, and 8.8 mm was used for eyes with no astigmatism. Each ablation was performed using the WaveLight^®^ EX500 excimer laser system. An optic zone of 6.5 mm with a total ablation zone of between 7.1 to 9.0 mm was used. The total ablation zone was calculated based on preoperative scotopic pupil size. Wavefront-optimized treatment profiles were used for all patients with emmetropia being the target refraction. Postoperatively, patients were prescribed ofloxacin 1% (Ocuflox, Alcon Labs, Ft Worth, TX, USA) and dexamethasone 0.1% (Maxidex, Alcon Labs, Ft Worth, TX, USA) eye drops to be taken 4 times a day for one week and then 3 times a day for a second week along with artificial tears. Patients were then advised to avoid strenuous activities such as sports for 6 weeks and to avoid contact sports indefinitely following the surgery.

### 2.4 Postoperative Assessment

Vision and slit-lamp examination were assessed at day 1 postoperatively. Further assessments were held at 1 month and 3 months. Postoperative assessments included visual acuity (UDVA and CDVA, logMAR), subjective refraction, contrast sensitivity, topography, assessment of HOA and quality of vision questionnaires.

### 2.5 Statistical Analysis

Mean and the standard deviation was calculated for continuous variables and significance was tested using paired two-tailed t-tests assuming unequal variance. Correlations between variables were tested by performing a Pearson correlation coefficient test. A p-value less than 0.05 was considered statistically significant.

### 2.6 Consent

Prior to surgery, each patient was informed about the surgery, potential outcomes and possible complications. In accordance with the ethics requirements and good clinical practice, each subject signed an informed consent form before proceeding with surgery. Written consent of participation and publication for the study was also obtained prior to surgery.

## 3 RESULTS

All patients attended the scheduled follow-up visits.

Refractive, visual acuity and aberration data are summarised in Table **1**.

### 3.1 Refraction

**Fig. (1):**
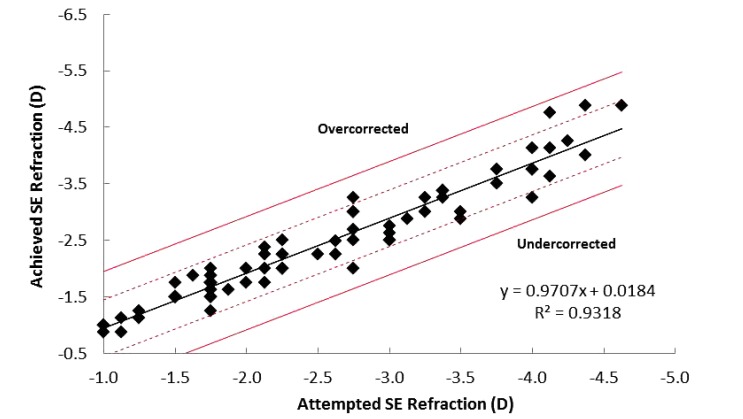
Achieved versus attempted SE. The black linear line represents the results of the linear regression analysis. The solid red line indicates error margin of ±1.0 D and dashed red line indicates error margin of ±0.5 D.

Mean postoperative SE was reduced to -0.06 ± 0.3 D at 1 month postoperatively, and with a further non-significant decrease by 3 months (-0.09 ± 0.26 D). Achieved SE was within ± 0.5 D of the attempted SE for 95% of patients, with 100% of patients being within ± 1.0 D (Fig. **1**). Assessment of attempted vs. achieved SE outcomes suggested a trend of slight under-correction with a correction ratio of 0.9318 (Fig. **1**).

**Fig. (2):**
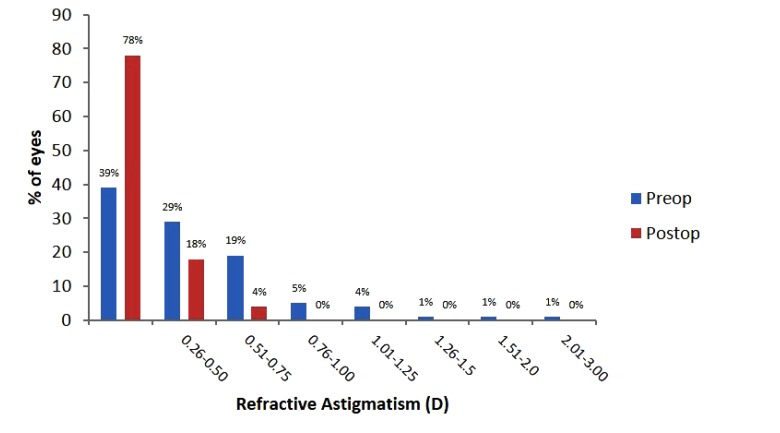
Percentage of patients with refractive astigmatism within each range preoperatively and at three months postoperatively.

Prior to surgery, 68% of patients had astigmatism of ≤0.5 D (Fig. **2**). Average postoperative refractive astigmatism was 0.17 ± 0.23 D at 1 month and 0.16 ± 0.23 D at 3 months postoperatively, with 96% of patients being ≤0.5 D (Fig. **2**).

### 3.2 Visual Acuity

At 3 months postoperatively, average UDVA was -0.08 ± 0.07 logMAR, with 96.1% of patients having UDVA of 20/20 or better, 60% of 20/16 or better and 10% of 20/12.5 or better. No patients reported postoperative UDVA of 20/32 or worse. Average postoperative CDVA was -0.13 ± 0.06 logMAR, being equal to or better than preoperative CDVA for 96% of patients, with 20% of patients reporting no change, 62% reporting a gain of 1 line and 14% reporting a gain of ≥2 lines. No patients reported a loss of ≥2 lines. While 3% of patients reported a loss of 1 line, all of these patients maintained CDVA of 20/20 or better.

### 3.3 Contrast Sensitivity

**Fig. (3):**
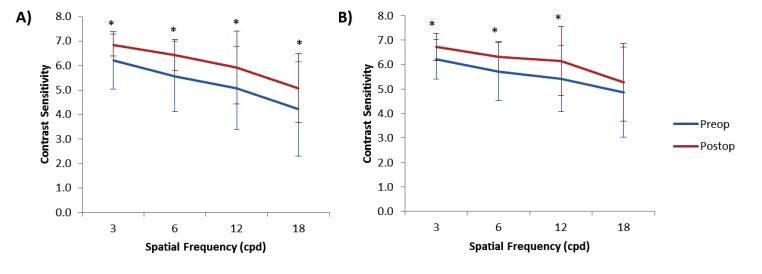
CS against (**A**) horizontal gratings and (**B**) vertical gratings at each spatial frequency. Data reported as mean ± 1 standard deviation. Significance (P≤0.05) is indicated by an asterisk.

Postoperative CS against both horizontal and vertical gratings increased compared to preoperative values at all spatial frequencies with the exception of 18 cycles per degree (cpd) for vertical gratings (Figs. **3a** and **3b**). Postoperative CS against horizontal gratings increased from 6.21 ± 1.17 to 6.84 ± 0.44 at 3 cpd (P = 0.003), from 5.55 ± 1.45 to 6.42 ± 0.64 at 6 cpd (P = 0.001), from 5.08 ± 1.70 to 5.92 ± 1.50 at 12 cpd (P = 0.02) and from 4.24 ± 1.92 to 5.08 ± 1.40 at 18 cpd (P = 0.03) (Fig. **3a**). Postoperative CS against vertical gratings increased from 6.21 ± 0.81 to 6.71 ± 0.57 at 3 cpd (P = 0.002), from 5.71 ± 1.18 to 6.32 ± 0.62 at 6 cpd (P = 0.007), and from 5.42 ± 1.35 to 6.13 ± 1.42 at 12 cpd (P = 0.03) (Fig. **3b**). There was no significant change in contrast sensitivity against vertical gratings at 18 cpd (Fig. **3b**).

### 3.4 HOAs

**Fig. (4):**
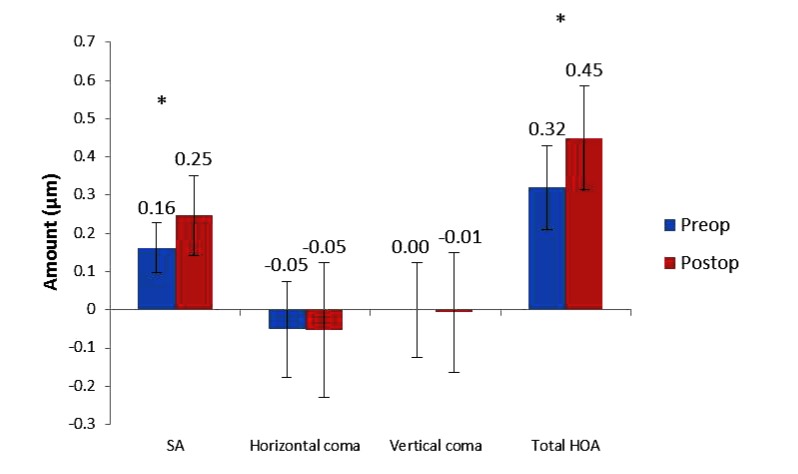
Higher order aberrations preoperatively and at 3 months postoperative. Data presented as mean ± 1 standard deviation. Significance (P≤0.5) is indicated by an asterisk.

Average SA increased by 52% from 0.162 ± 0.065 µm preoperatively to 0.247 ± 0.105 µm at 3 months postoperatively (*P* = 0.000) and total HOA RMS increased by 41% from 0.320 ± 0.109 µm preoperatively to 0.450 ± 0.136 µm at 3 months postoperatively (P = 0.000) (Fig. **4**). There was no significant change in horizontal or vertical coma (Fig. **4**).

### 3.5 VF14 QOL Questionnaire

Subjective quality of vision increased from 89.2 ± 16.7% to 99.0 ± 1.4% at 3 months postoperatively (*p* = 0.001).

### 3.6 Correlations

Postoperative SA positively correlated with postoperative HOA (r = 0.414, P = 0.000) and postoperative SE negatively correlated with total VF14 score (r = -0.324, P = 0.004). Postoperative UDVA negatively correlated with postoperative SE (r = -0.292, P = 0.011), horizontal CS at 12 cpd (r = -0.461, P = 0.000) and 18 cpd (r = -0.489, P = 0.000), and vertical CS at 3 cpd (r = -0.295, P = 0.009) and 18 cpd (r = -0.439, P = 0.000) *i.e.* contrast sensitivity improved as UDVA improved. There was a strong negative correlation between postoperative UDVA and postoperative vertical CS at 12 cpd (r = -0.586, P = 0.000). There was no correlation between postoperative SA or HOAs with CS, or postoperative SE with total postoperative VF14 score.

## 4 DISCUSSION

The introduction of HOAs is a common complication of corneal refractive procedures. The development of femtosecond lasers and the introduction of wavefront ablation systems help to minimise the induction of HOAs, but results are often variable and not always notably superior to conventional systems. This study performed LASIK using a wavefront-optimised laser platform with eye tracking (*i.e.* WaveLight^®^ FS200 Femtosecond and wavefront-optimised EX500 excimer laser platform) to assess the outcomes of the procedure on low-myopic astigmatic eyes.

The introduction of optical aberrations correlates with visual complaints following refractive corneal surgery [11]. Several mechanisms may explain the induction of HOAs including irregular astigmatism, a more oblate corneal shape, decentration and a smaller optical zone as well as larger pupil size [12]. Wavefront ablation systems were introduced in an attempt to minimise the HOAs that were introduced *via* conventional ablation. While some studies have reported that wavefront technology provides better UDVA and contrast sensitivity compared to conventional ablation [13, 14], others have found the relative advantage to be dependent on what laser platform is used [15]. A meta-study analysis of 65 papers comparing wavefront-optimised to conventional ablation also indicated improved UDVA, contrast sensitivity and accuracy, with less residual refractive error and visual symptoms following wavefront-guided compared to conventional ablation [16]. When the Allegretto excimer laser was used to perform wavefront-guided or wavefront–optimised ablation, it was reported that wavefront-guided ablation provided better VA, contrast sensitivity, SE, accuracy, trefoil and HOA induction and residual astigmatism compared to optimised ablation [17, 18].

Mean postoperative SE (*i.e.* -0.09 ± 0.26 D), refractive astigmatism (*i.e.* -0.15 ± 0.24 D; Fig. **2**) and UDVA (-0.08 ± 0.08 logMAR) for this study were similar or better than that reported for eyes with comparable preoperative SE following wavefront-guided [19, 20] and wavefront-optimised ablation [19-21] on different laser platforms. He *et al.* [13] previously reported reduced refractive stability following wavefront-guided ablation compared to wavefront–optimised, with SE becoming more negative within 3 months following wavefront-optimised ablation. Unfortunately, long-term stability could not be measured for this study due to the shorter follow-up time, however, SE remained stable up to 3 months following the procedure. It has been previously suggested that wavefront-guided ablation is best suited for people with high levels of preoperative HOAs *i.e.* ≥ 0.35 µm due to improved refractive outcome and stability [14, 17]. The mean preoperative HOA RMS for this study was 0.32 µm. Although the results are consistent with previous reports suggesting that wavefront-optimised ablation is suitable for patients with this level of preoperative HOA, it cannot be determined from these results whether wavefront-guided ablation would provide better postoperative outcomes for these patients.

This study reports an increase in total HOAs of 41% and in SA of 52%. Although some studies have reported no significant change in HOAs compared to preoperative levels following wavefront-optimised and wavefront-guided ablation using the VISX26 [22] and Allegretto [19] platforms respectively, reports are mixed with some studies finding that HOAs can be increased by LASIK. The increases in total HOAs reported for this study are lower than that reported previously following wavefront-guided LASIK on eyes with comparable preoperative SE, with Ganesh and Gupta [23] reporting an increase of 66% in HOAS following LASIK utilising the Schwind laser platform. Miraftab *et al.* [24] also reported an increase of 73% following wavefront-optimised and 61% following wavefront–guided ablation of eyes with moderate preoperative SE using the Allegretto Concerto laser [24]. As the amount of HOA induction strongly corresponds with both preoperative myopia [25] and astigmatism [26], this larger increase in HOAs reported by Miraftab *et al.* [24] is likely due to the higher preoperative astigmatism. The relatively low amount of induced HOAs for this study is likely due to a combination of factors. Firstly, optimised ablation induces less HOAs than conventional ablation and similar amounts to guided ablation while eye tracking technology reduces decentration to further reduce the induction of coma. Secondly, the use of femtosecond lasers for flap formation is shown to reduce HOA induction, particularly of SA and lead to better postoperative CS compared to mechanical microkeratomes [27]. Finally, as the amount of induced HOAs corresponds strongly to both preoperative myopia [25] and astigmatism [26], the low preoperative sphere and cylinder values of the patients in this cohort would have contributed to low postoperative values. Despite HOA values increasing as is commonly reported, the amount of postoperative HOAs did not correlate with changes in subjective vision or UDVA, suggesting that the amount of induced HOAs was not sufficient to affect vision or patient satisfaction.

Reports regarding the effect of corneal refractive procedures on postoperative contrast sensitivity remain conflicting [28-30]. However, the type of ablation utilised appears to be a factor, with reports of improved contrast sensitivity following wavefront-guided ablation [14, 31, 32], but decreased contrast sensitivity following conventional ablation [14]. This decrease, however, is generally temporary with contrast sensitivity returning to normal levels within 3-12 months [14, 23, 29, 33]. For this study, contrast sensitivity increased postoperatively for both horizontal (Fig. **3a**) and vertical gratings at all spatial frequencies with the exception of vertical gratings at 18 cpd (Fig. **3b**) despite an increase in HOAs and remained so at 3 months postop. This outcome is likely due to the fast speed of the excimer laser used as well as the eye-tracking technology, which would have helped to reduce decentration, thereby preventing the induction of coma.

Subjective vision following LASIK has been reported to be dependent on preoperative expectations, psychological characteristics, visual function, UDVA achieved [34] and residual refractive error [35]. Tasks for which patients reported having the most difficulty both pre- and postoperatively included nighttime driving, reading signs and daytime driving. Although this study did not evaluate visual artefacts, the difficulty experienced for these tasks was likely due to the presence of glare and haloes, both of which have been previously documented [23, 36]. Nevertheless, an improvement in the subjective quality of vision was achieved with lower levels of difficulty reported postoperatively for all tasks. Additionally, reduced residual refractive error corresponded with improved postoperative UDVA and a weak correlation between postoperative UDVA and subjective vision is reported, although the results did not reach statistical significance (r = -0.052, P = 0.65).

Small Incision Lenticule Extraction (SMILE) is a flap-free procedure first developed to treat myopia. In a recent meta-study analysis that compared outcomes of FS-LASIK or SMILE, it was reported that SMILE offers little benefit in terms of postoperative CDVA or refractive accuracy [37]. However, LASIK treatment resulted in a higher incidence of postoperative dry eye and loss of corneal sensitivity [37]. Many studies that have compared SMILE and LASIK for the correction of low astigmatism used cohorts with a moderately myopic preoperative sphere *i.e.* 2.0-5.0 D [23, 38, 39]. The current study addresses this gap in the literature by recruiting a cohort with low-astigmatism (mean preoperative cylinder of -0.47 D) and low-myopia (mean preoperative sphere of -2.26 D). The results of this study could not be compared directly with a SMILE-treatment cohort due to time restraints. However, the current study reports a postoperative SE closer to emmetropia (-0.09 versus -0.14 respectively), and a higher proportion of patients achieving 20/20 CDVA or better (96% versus 84% respectively) than what has previously been reported for low-astigmatic patients treated with SMILE [23]. This difference is likely due to the laser platforms; this study utilised the Allegretto WaveLight excimer laser for corneal ablation while the previous study utilised a Schwind laser [23]. When comparing the use of the WaveLight Allegretto and the Schwind lasers for LASIK on myopic astigmatic patients, Bohac *et al.* reported a 0.5 line improvement in postoperative UDVA compared to preoperative CDVA when the WaveLight Allegretto laser was used, while no improvement was observed when the Schwind laser was used [40].

It has also been reported that SMILE induces less topographical changes compared to LASIK, although an increase in a vertical coma has been reported [38] likely due to the reliance on subjective fixation rather than eye tracking technology. This study reports no increase in a vertical or horizontal coma following LASIK which is consistent with the use of eye-tracking technology. Additionally, this study reports a more modest increase in total HOAs than what has been reported for a low-astigmatic cohort following SMILE; Chen *et al.* reported a 61% increase in HOAs [38] compared to 41% reported for this study.

The limitations of this study include a small sample size, short follow up time and the use of only a single laser platform for comparison against previous publications. Additionally, common visual artefacts such as haloes and glare which may affect patient satisfaction were not evaluated so could not be taken into account when reporting subjective vision.

## CONCLUSION

For this study, postoperative visual acuity, CS and subjective vision were dependent on postoperative SE and not affected by the level of HOAs. Good visual and refractive outcomes were achieved, comparable to those achieved following wavefront-guided ablation without the decrease in contrast sensitivity and lower stability and accuracy of SE previously associated with wavefront-optimised ablation. While newer technologies such as SMILE have become available for patients who require more extensive treatment such as high myopes, these results indicate that LASIK performed using this laser platform with wavefront-optimised ablation is still an excellent option for treatment of low-myopic astigmatic eyes.

## Figures and Tables

**Table 1:** Ocular parameters preoperatively and at 1 and 3 months postoperative.

–	**Preop**	**1 Month Postop**	**3 Month Postop**	**P Value**
**Sphere (D)**	-2.26 ± 0.95	0.01 ± 0.32	-0.04 ± 0.28	0.00
**Cylinder (D)**	-0.47 ± 0.46	-0.18 ± 0.23	-0.15 ± 0.24	0.00
**SE (D)**	-2.49 ± 1.0	-0.06 ± 0.31	-0.09 ± 0.26	0.00
**CDVA (LogMAR)**	-0.03 ± 0.09	-0.11 ± 0.06	-0.13 ± 0.06	0.00
**SA (μm)**	0.16 ± 0.06	0.24 ± 0.10	0.25 ± 0.10	0.00
**Horizontal coma (μm)**	-0.05 ± 0.13	-0.03 ± 0.21	-0.05 ± 0.18	0.46
**Vertical coma (μm)**	0.00 ± 0.12	-0.01 ± 0.15	-0.01 ± 0.16	0.36
**HOA RMS (μm)**	0.32 ± 0.11	0.44 ± 0.13	0.45 ± 0.14	0.00

SE = spherical equivalent; CDVA = corrected distance visual acuity; SA = spherical aberration; HOA RMS = higher order aberrations root mean square.

## LIST OF ABBREVIATIONS

CDVA: = Corrected Distance Visual Acuity
CPD: = Cycles Per Degree
CS: = Contrast Sensitivity
HOA: = Higher Order Aberration
LASIK: = Laser-Assisted *Situ In* Keratomileusis
RMS: = Root Mean Square
SA: = Spherical Aberration
SE: = Spherical Equivalent
SMILE: = Small Incision Lenticule Extraction
UDVA: = Unassisted Distance Visual Acuity
